# Supplementary material for: Macrophage deficiency of miR‐21 promotes apoptosis, plaque necrosis, and vascular inflammation during atherogenesis
Source: EMBO Mol Med. 2017 Jul 3;9(9):1244–62. doi: 10.15252/emmm.201607492 (PMC5582411; doi:10.15252/emmm.201607492)

Appendix table of Content

1. **Appendix Figure Legends S1-4**
2. **Appendix Figures S1-4**

SUPPLEMENTAL FIGURE LEGENDS

Fig S1. miR-21 levels in monocytes, neutrophils and T cells isolated from blood of WT mice.

Representative contour plots demonstrate gating scheme for sorted blood cells. Monocytes ($CD45^+/CD11b^+/CD115^+$), neutrophils ($CD45^+/CD11b^+/CD115^-$) and T Cells ($CD45^+/CD3^+$). Graph on the bottom right is the relative miR-21 expression levels normalized to U6 of the indicated cells types sorted from blood. Data are mean of triplicates (n=3; * *P <0.018* and # *P <0.0013* compared to monocytes/macrophages)

Fig S2. Absence of miR-21 in hematopoietic cells does not influence body weight and circulating lipids.

A-F) Measurement of body weight (**A**), plasma cholesterol (**B**), HDL-cholesterol (**C**), triglyceride (**D**), and lipoprotein profile from pooled plasma (**E** and **F**) of $Ldlr^{-/-}$ and $Ldlr^{-/-}miR\text{-}21^{-/-}$ mice before and after 12 weeks on WD diet. Data are average (n=10 per group).

Fig S3. Genetic ablation of miR-21 in hematopoietic cells does not influence macrophage proliferation in atherosclerotic lesions.

Quantification of Ki67/CD68 positive macrophages in plaques isolated from $Ldlr^{-/-}$ mice transplanted with WT and $miR\text{-}21^{-/-}$ BM. Data are average (n=10 per group).

Fig S4. Effect of apoptotic stressors on in macrophage deficient of miR-21.

Western blot analysis of PTEN, p-AKT, AKT and MKP1 of WT or $miR\text{-}21^{-/-}$ peritoneal macrophages treated with Tunicamycin (5µg/ml), FC load = with Ac-LDL (120 µg cholesterol /ml) + ACAT inhibitor [Sandoz 58035 (10mg/ml)] or Thapsigargin (2µM) for the indicated times. HSP90 is a loading control.

Appendix Figure S1

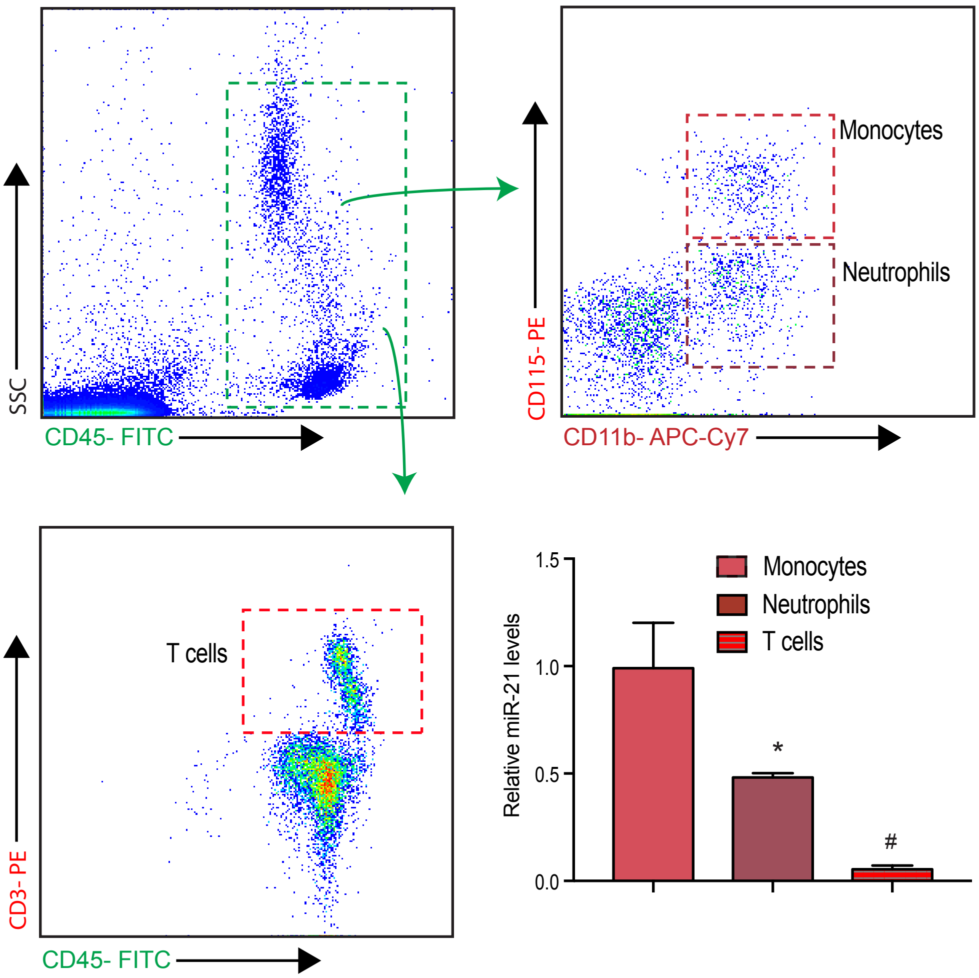

Appendix Figure S2

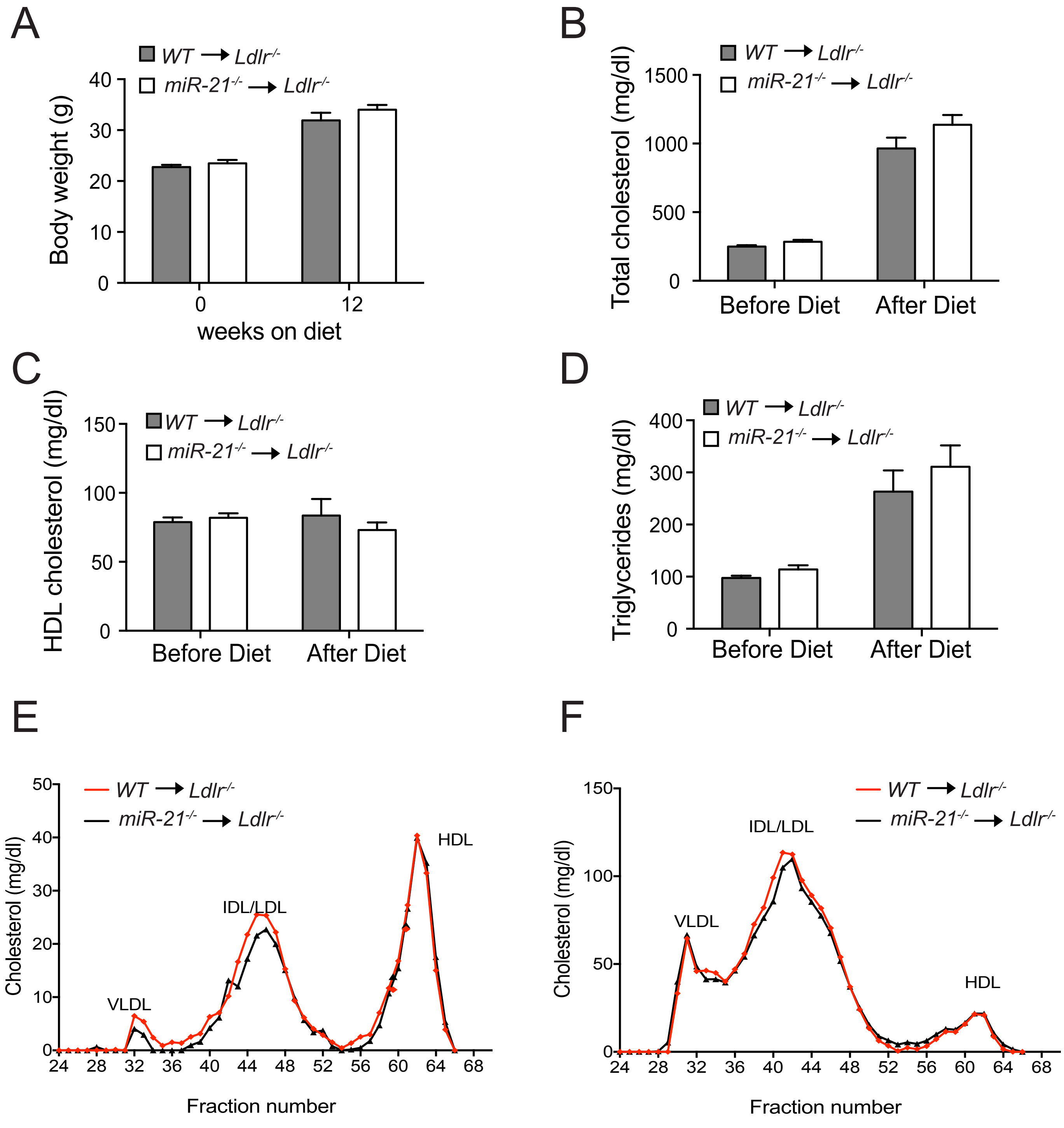

Appendix Figure S3

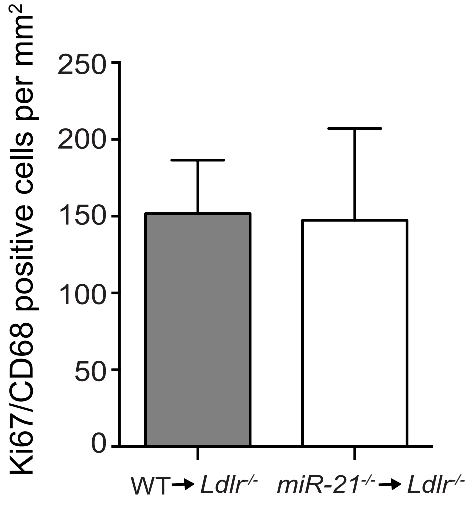

Appendix Figure S4

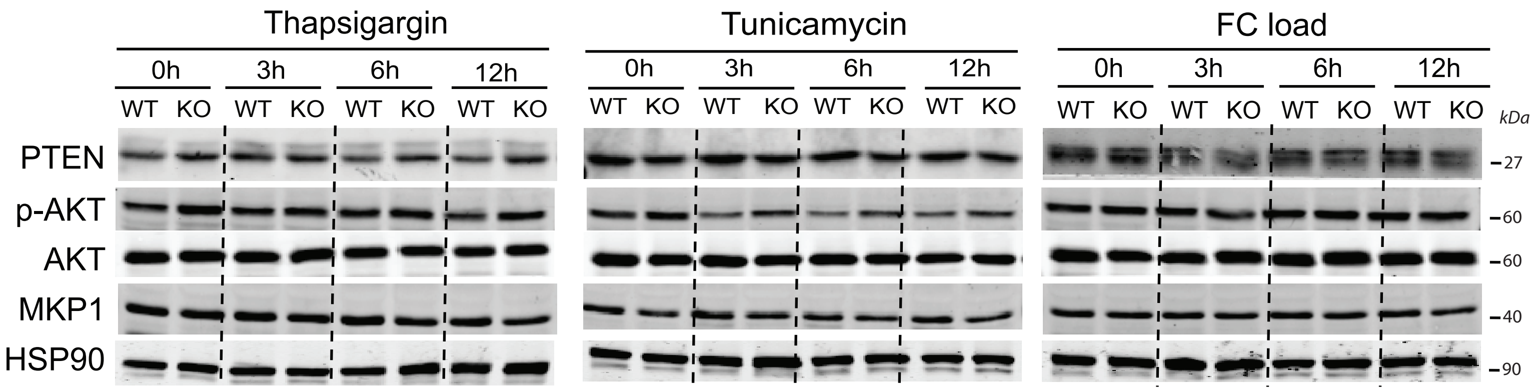

Supplement: Supplementary file 1 — Appendix [file EMMM-9-1244-s001.pdf]
