# Supplementary figures and images for: Macrophage deficiency of miR‐21 promotes apoptosis, plaque necrosis, and vascular inflammation during atherogenesis
Source: EMBO Mol Med. 2017 Jul 3;9(9):1244–62. doi: 10.15252/emmm.201607492 (PMC5582411; doi:10.15252/emmm.201607492)

## Original Western blots from Figure S 4

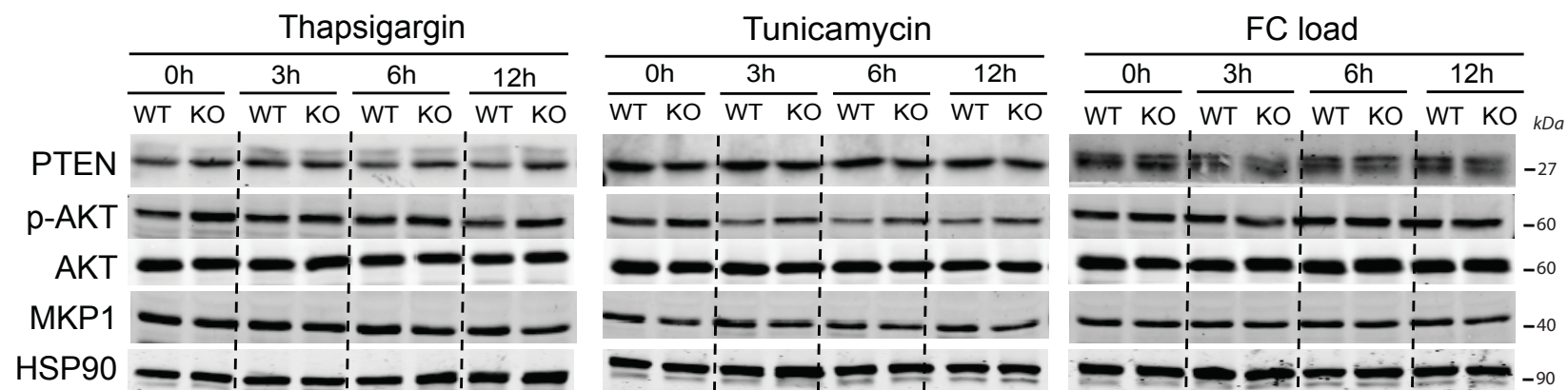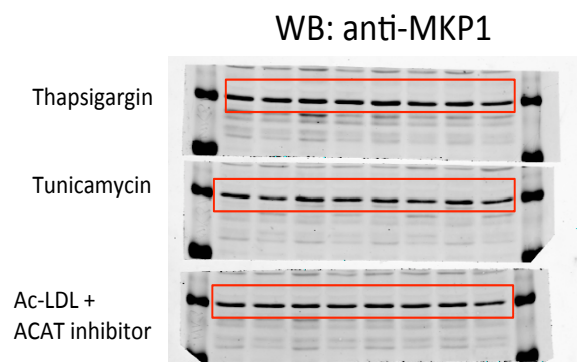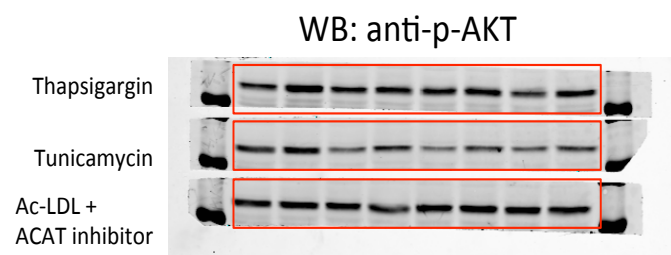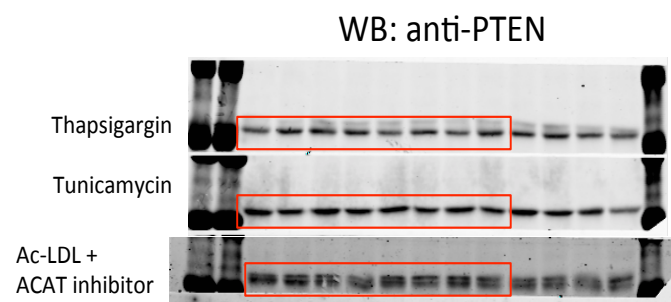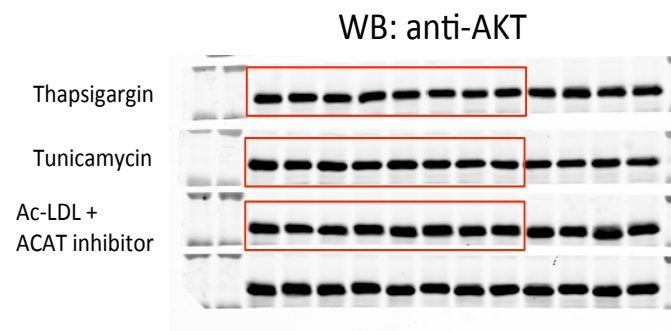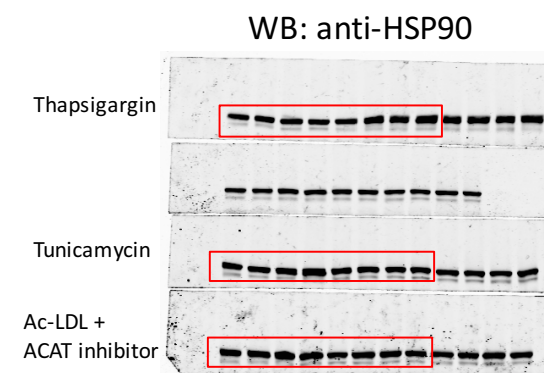

Supplement: Supplementary file 2 — Source Data for Appendix [file EMMM-9-1244-s002.zip › SData_Appendix/Source_data_Figure_S4.pdf]

## Original Western blots from Figure 4 E

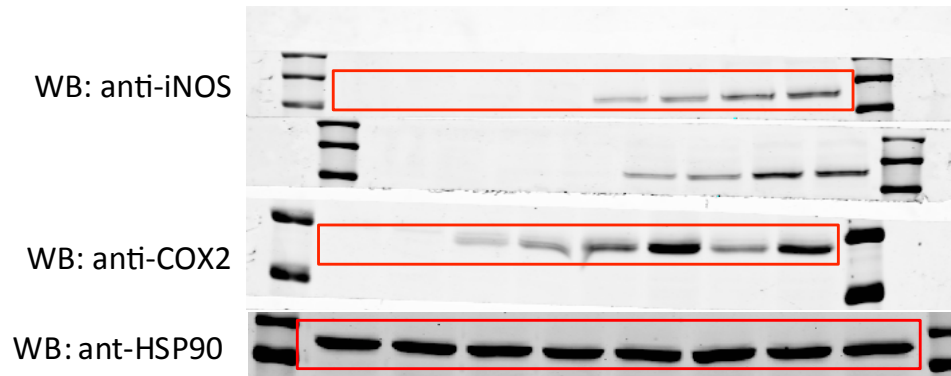

E

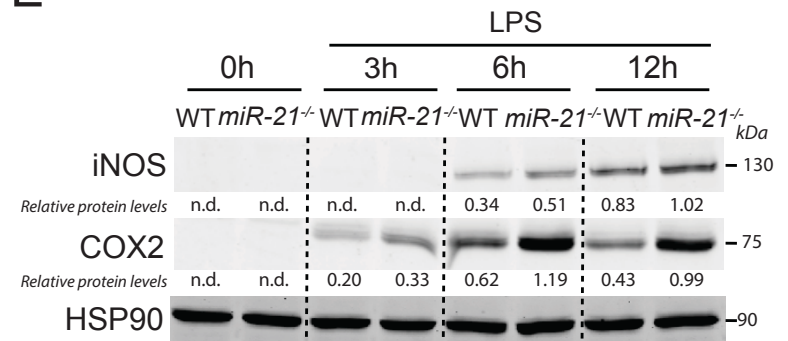

Supplement: Supplementary file 4 — Source Data for Figure 4 [file EMMM-9-1244-s003.pdf]
