# Supplementary material for: Macrophage deficiency of miR‐21 promotes apoptosis, plaque necrosis, and vascular inflammation during atherogenesis
Source: EMBO Mol Med. 2017 Jul 3;9(9):1244–62. doi: 10.15252/emmm.201607492 (PMC5582411; doi:10.15252/emmm.201607492)

Original Western blots from Figure 5 B

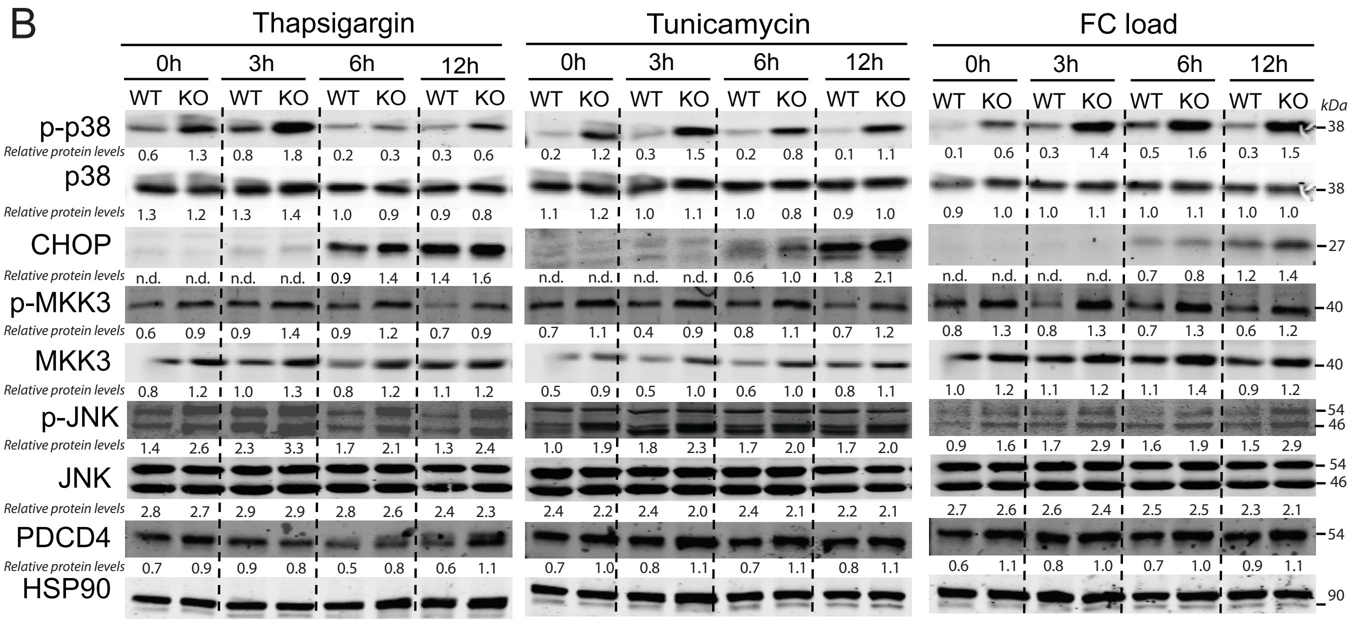

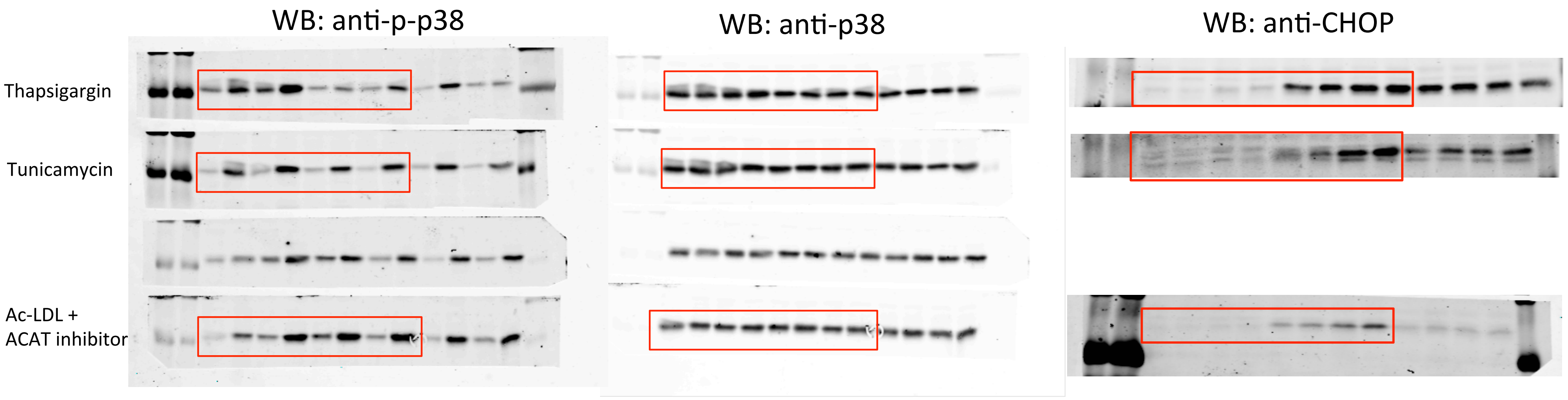

Original Western blots from Figure 5 B

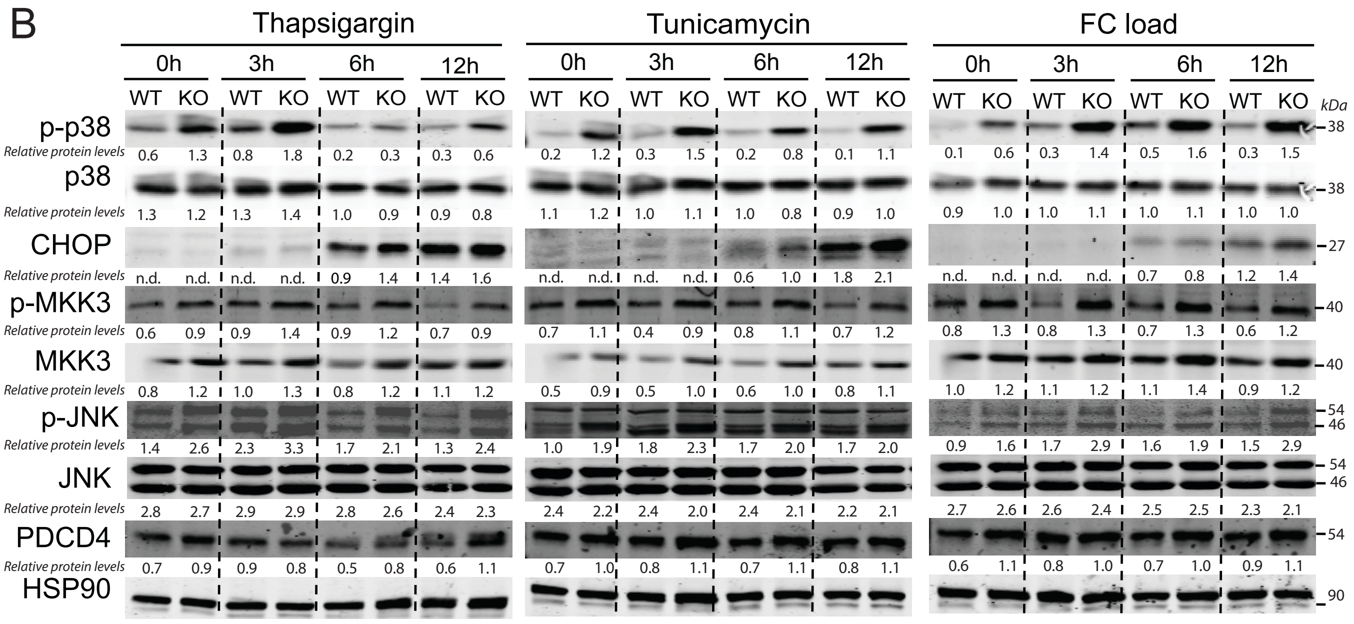

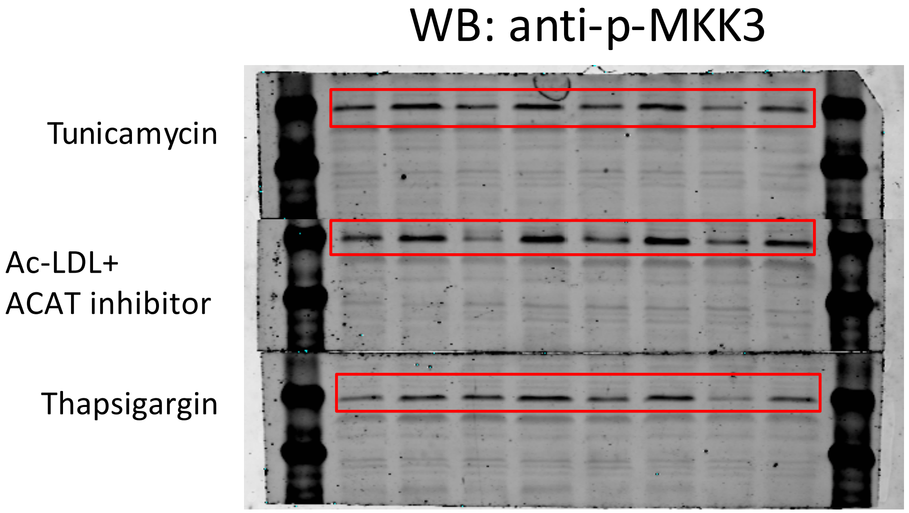

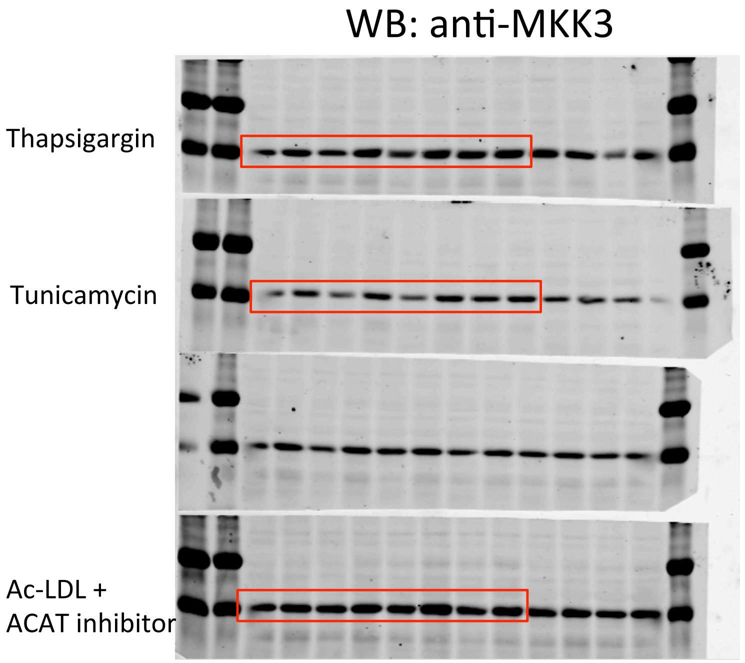

Original Western blots from Figure 5 B

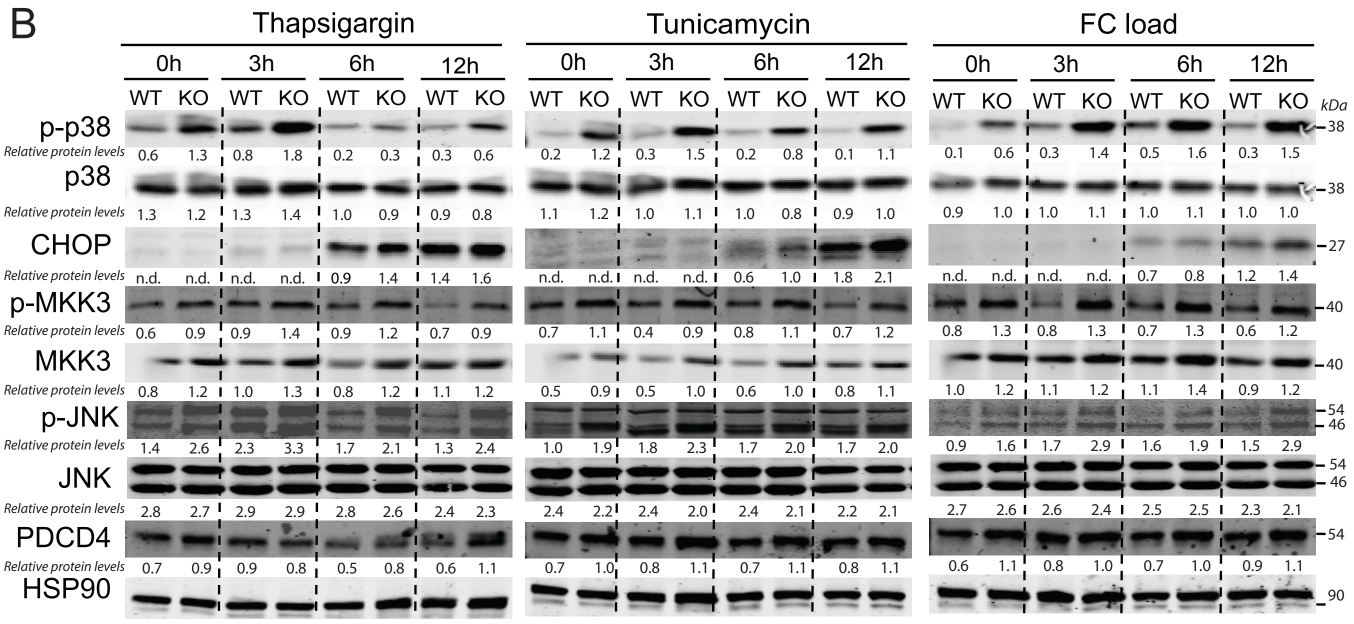

WB: anti-p-JNK

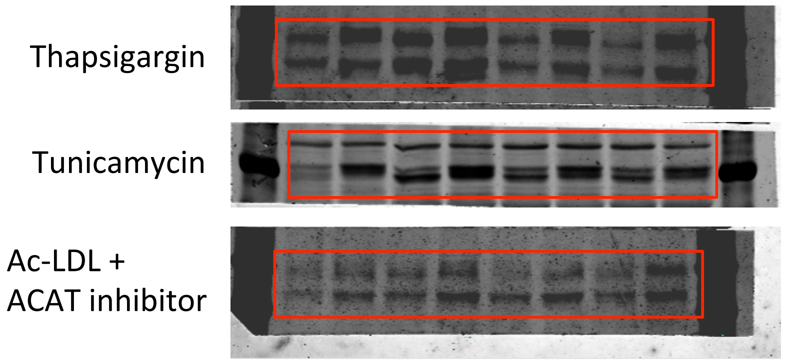

WB: anti-JNK

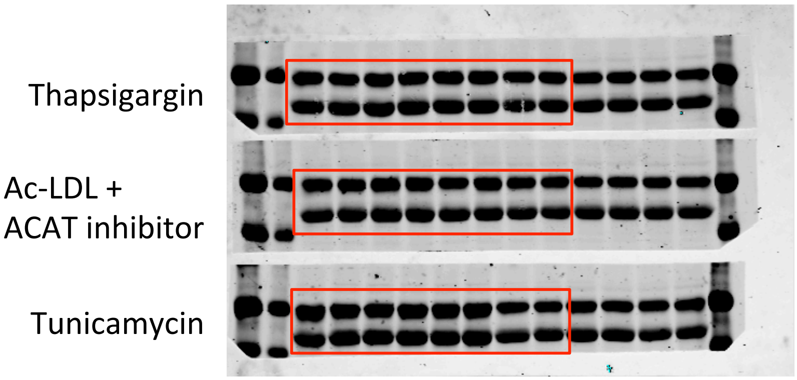

Original Western blots from Figure 5 B

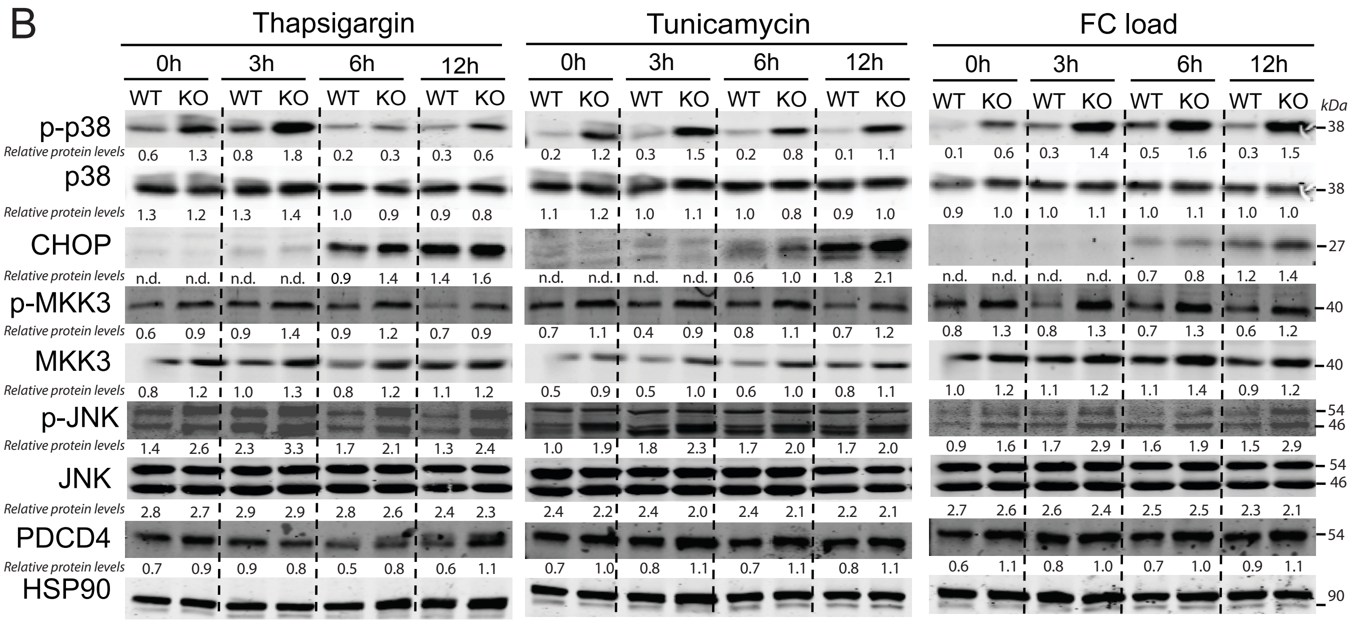

WB: anti-PDCD4

Thapsigargin

Tunicamycin

Ac-LDL +
ACAT inhibitor

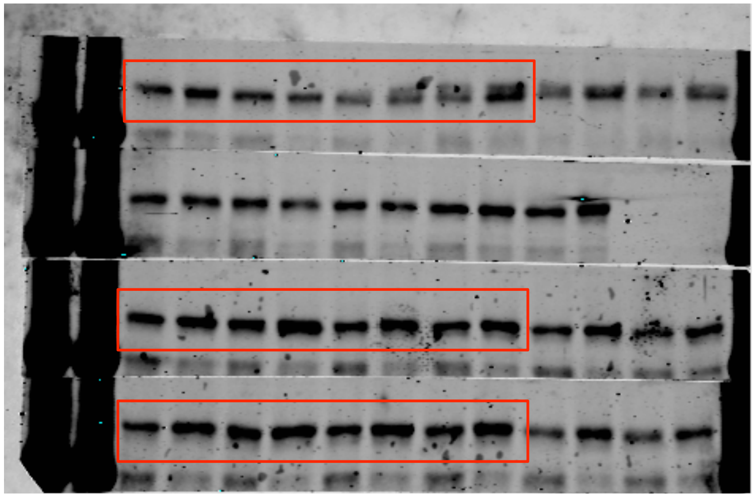

WB: anti-HSP90

Thapsigargin

Tunicamycin

Ac-LDL +
ACAT inhibitor

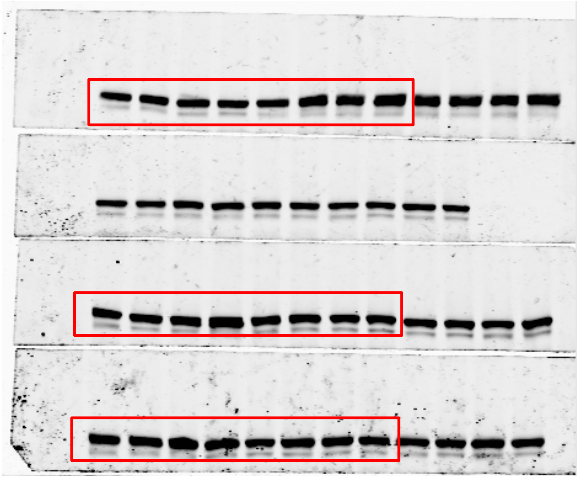

Original Western blots from Figure 5 C

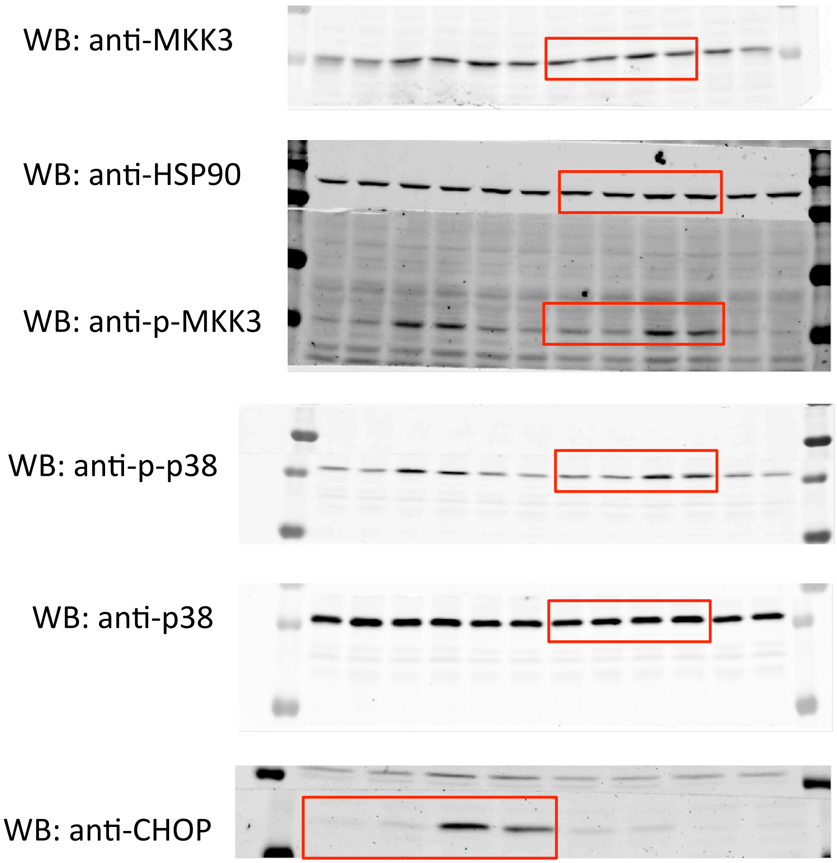

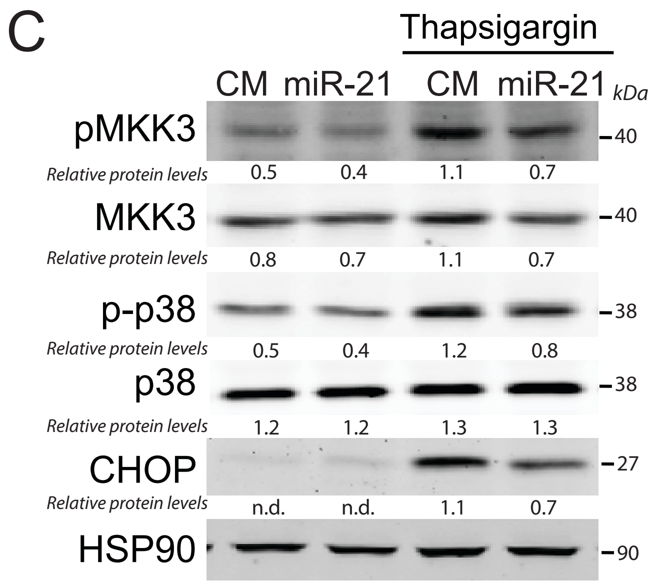

Original Western blots from Figure 5 D

WB: anti-CHOP

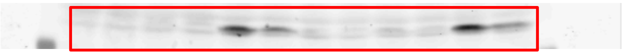

WB: anti-p-p38

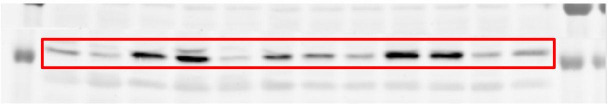

WB: anti-p38

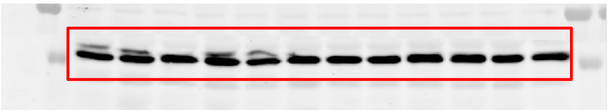

WB: anti-HSP90

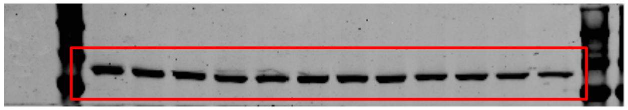

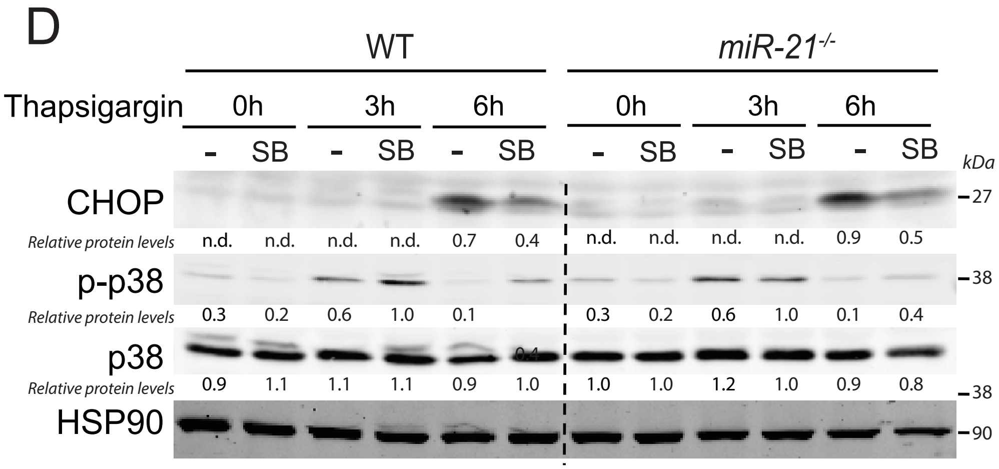

Supplement: Supplementary file 5 — Source Data for Figure 5 [file EMMM-9-1244-s004.pdf]
