# Supplementary material for: Macrophage deficiency of miR‐21 promotes apoptosis, plaque necrosis, and vascular inflammation during atherogenesis
Source: EMBO Mol Med. 2017 Jul 3;9(9):1244–62. doi: 10.15252/emmm.201607492 (PMC5582411; doi:10.15252/emmm.201607492)

Original Western blots from Figure 6 B

WB: anti-MerTK

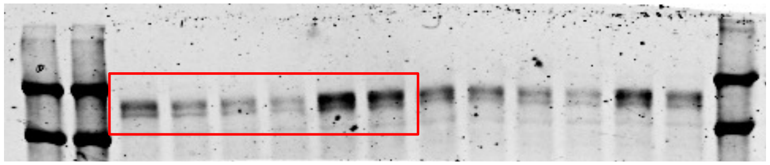

WB: anti-HSP90

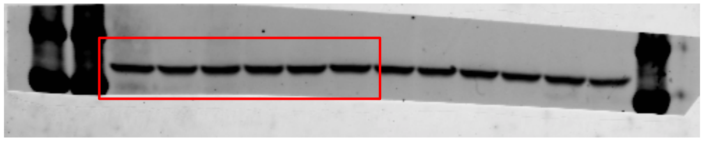

WB: anti-COX2

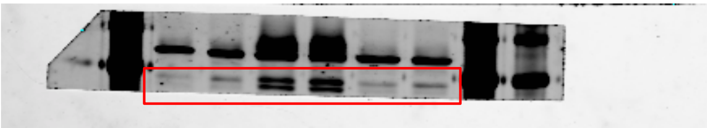

WB: anti-ABCA1

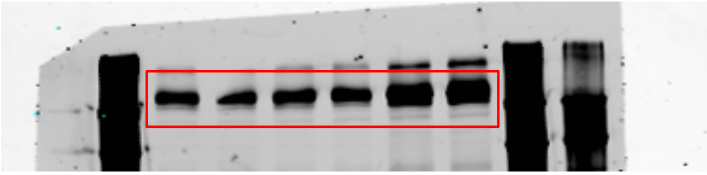

B

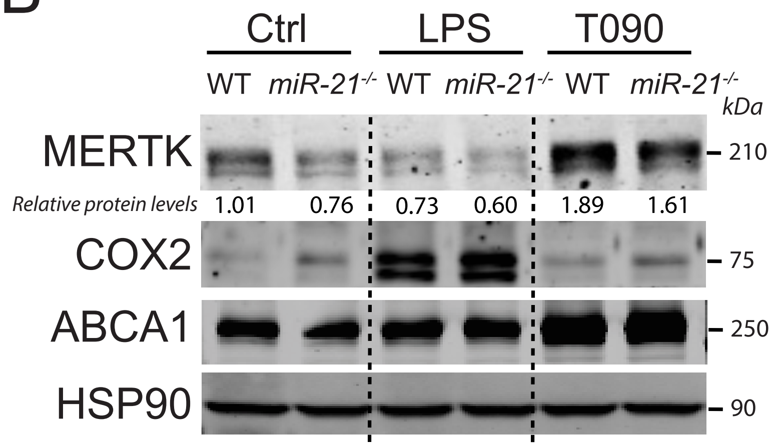

Original Western blots from Figure 6 D

WB: anti-MerTK

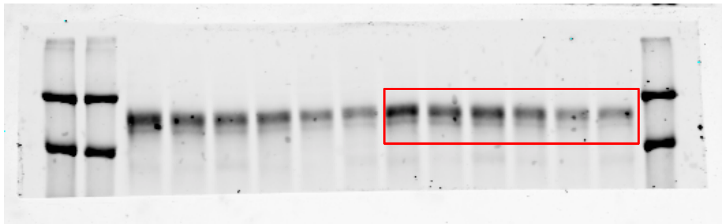

WB: anti-HSP90

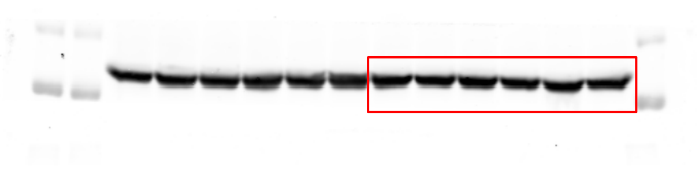

WB: anti-COX2

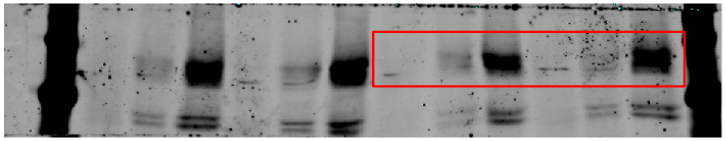

WB: anti-MerTK (soluble fraction)

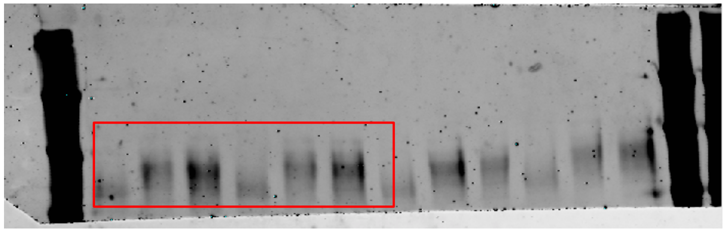

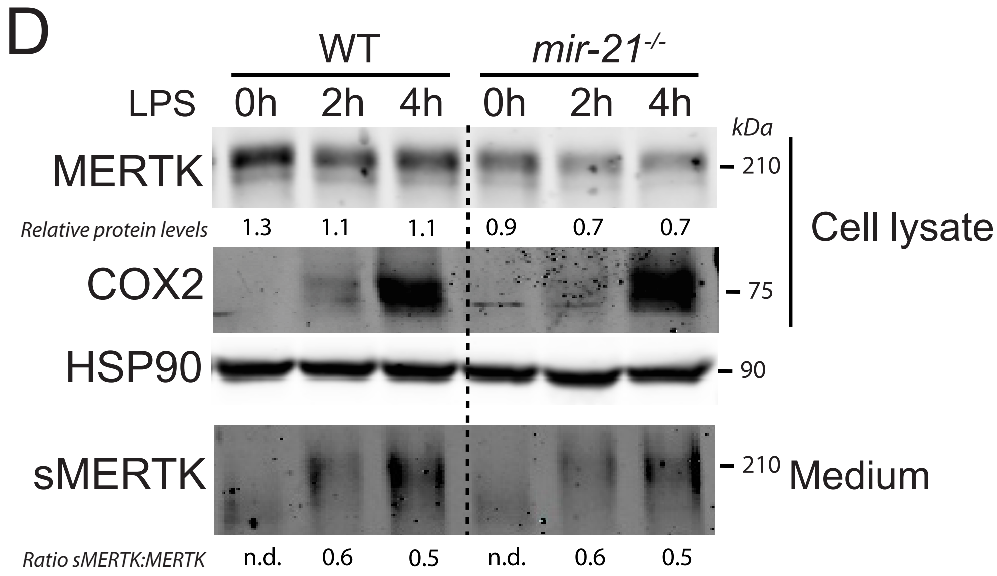

Supplement: Supplementary file 6 — Source Data for Figure 6 [file EMMM-9-1244-s005.pdf]
