# Supplementary material for: Macrophage deficiency of miR‐21 promotes apoptosis, plaque necrosis, and vascular inflammation during atherogenesis
Source: EMBO Mol Med. 2017 Jul 3;9(9):1244–62. doi: 10.15252/emmm.201607492 (PMC5582411; doi:10.15252/emmm.201607492)

Original Western blots from Figure 7 E

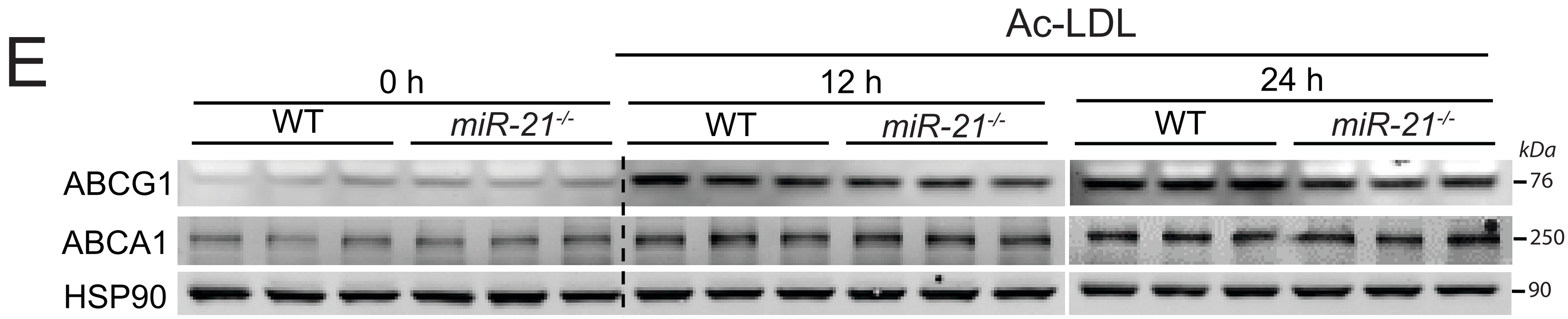

WB: anti-ABCA1

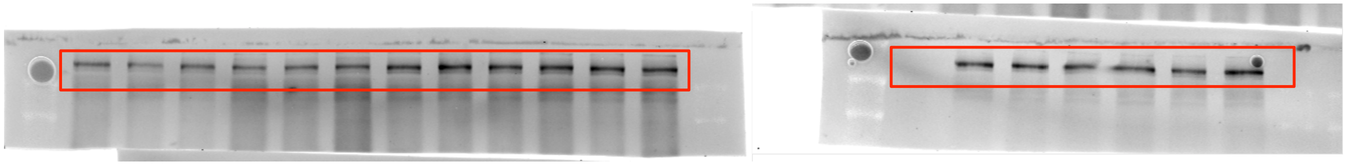

WB: anti-ABCG1

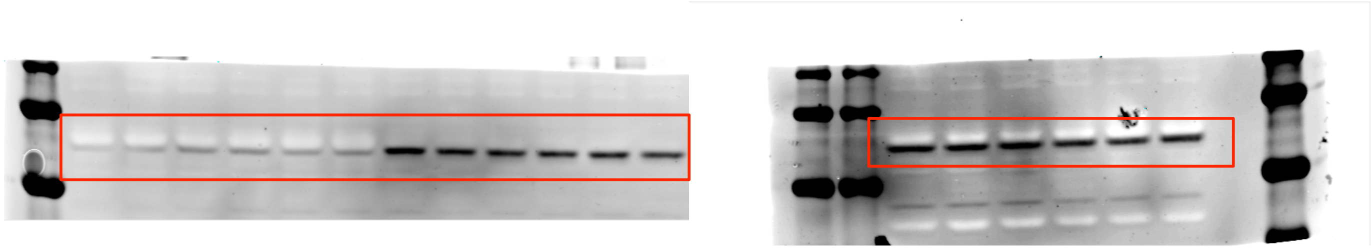

WB: anti-HSP90

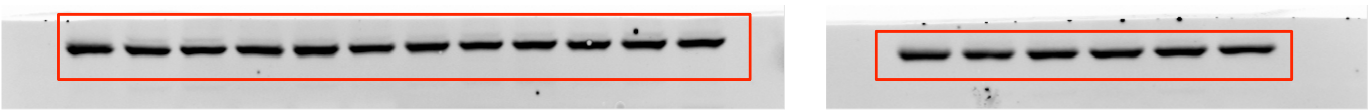

Original Western blots from Figure 7 F

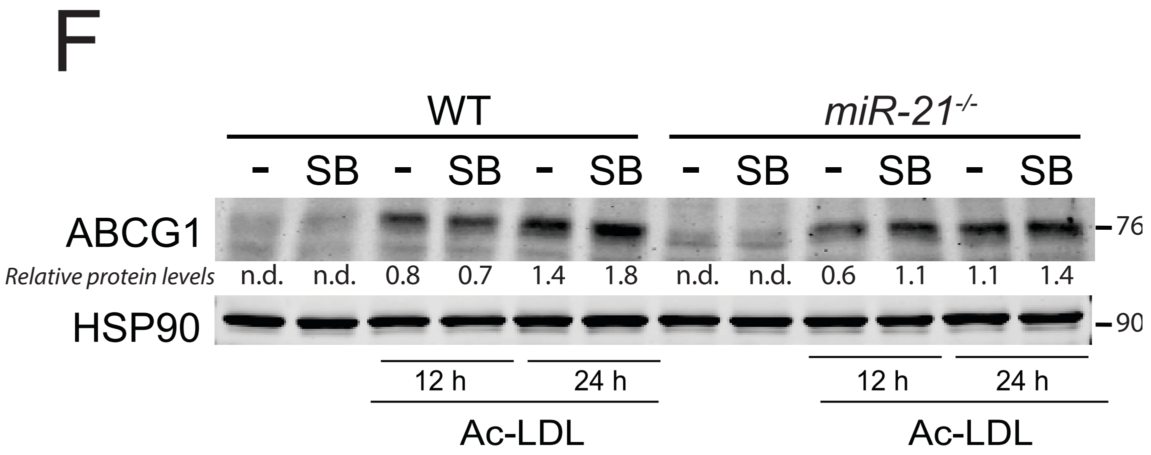

WB: anti-ABCG1

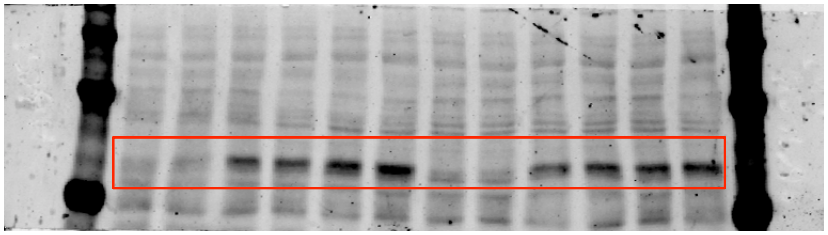

WB: anti-HSP90

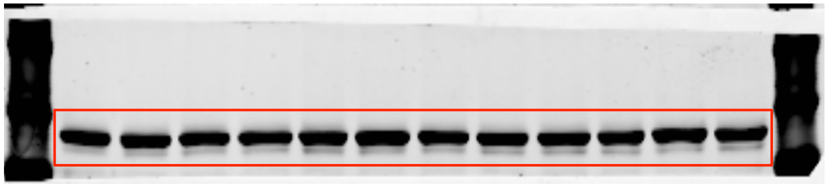

Original Western blots from Figure 7 G

G

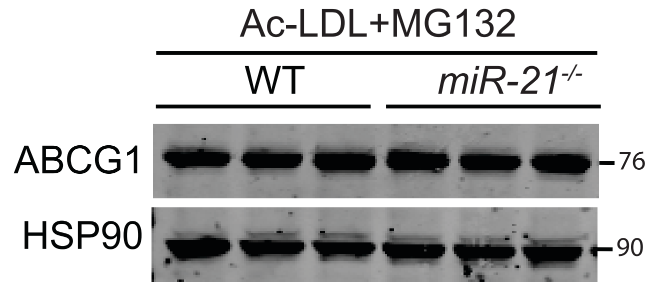

WB: anti-ABCG1

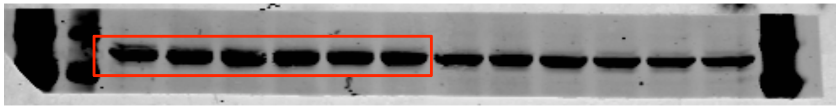

WB: anti-HSP90

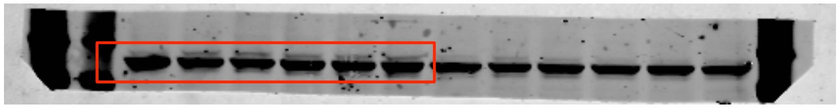

Supplement: Supplementary file 7 — Source Data for Figure 7 [file EMMM-9-1244-s006.pdf]
